# Inactivation of *Pseudomonas aeruginosa* and Methicillin-resistant *Staphylococcus aureus* in an open water system with ozone generated by a compact, atmospheric DBD plasma reactor

**DOI:** 10.1038/s41598-018-36003-0

**Published:** 2018-12-04

**Authors:** Bhaswati Choudhury, Sherlie Portugal, Navya Mastanaiah, Judith A. Johnson, Subrata Roy

**Affiliations:** 1 Applied Physics Research Group, University of Florida, 32611 Gainesville, USA; 2 Department of Mechanical and Aerospace Engineering, University of Florida, 32611 Gainesville, USA; 3 Department of Electrical and Computer Engineering, University of Florida, Gainesville, 32611 USA; 4 School of Electrical Engineering, Technological University of Panama, Panama City, Panama; 5 Department of Pathology, Immunology and Laboratory Medicine, University of Florida, Gainesville, 32611 USA; 6 Emerging Pathogens Institute, University of Florida, Gainesville, 32611 USA; 7 SurfPlasma, Inc., Gainesville, 32601 USA

## Abstract

Ozone is a well-known disinfecting agent that is used as an alternative for chlorine in many applications, including water decontamination. However, the utility of ozone in water decontamination is limited by high electrical power consumption and expensive, bulky equipment associated with ozone generation. This study investigates the effectiveness of a lightweight, compact surface dielectric barrier discharge (SDBD) reactor as an ozone generator to inactivate *Pseudomonas aeruginosa* and methicillin-resistant *Staphylococcus aureus* (MRSA) in an open water system. Experimental details are provided for ozone generation technique, mixing method, ozone concentrations in air and water, and input energy required to produce adequate ozone concentrations for bacterial inactivation in a contaminated, open water system. Specifically, an active plasma module (APM) reactor system of size 48 cubic centimeters, weighing 55 grams, with a maximum ozone yield of 68.6 g/KWh was used in atmospheric conditions as the source of ozone along with an air pump and a diffusion stone for mixing the ozone in water. Over 4-log reduction in *P. aeruginosa* concentration was achieved in 4 minutes with 0.1 mg/L ozone concentration in an open water system using 8.8 ± 1.48 J input energy. Also, over 5-log reduction in MRSA concentration was achieved in 2 minutes with 0.04 mg/L ozone concentration in an open water system using 4.4 ± 0.74 J input energy.

## Introduction

Despite major developments in water disinfection techniques over the years, water borne diseases continue to occur time after time^1^. During 2011–2012, there were 431 illnesses, including 14 fatal, related to drinking water and 1,788 illnesses involving recreational water, reported in the US^2,3^. Chlorination has been the most popular and accepted disinfection technique for drinking water as well as recreational water. However, chlorine is not completely effective against some microorganisms including *Giardia* cysts^4–9^. In addition, it reacts with organic matter to form a potentially carcinogenic group of chemicals called trihalomethanes (THMs)^10^. These drawbacks make chlorine less desirable as a disinfection agent.

Ozone is an attractive alternative for free chlorine and chloramine disinfection; with a higher thermodynamic oxidation potential, less sensitivity to organic material, and better tolerance for pH variations while retaining the ability to kill bacteria, fungi, viruses, as well as spores and cysts^11,12^. THM formation is also reduced by 98% with ozone treatment when compared to chlorine treatments^13^. Ozonation of water with a high concentration of bromide does lead to the formation of undesirable brominated disinfection byproducts, however, unless drinking water is produced by desalination, ozonation can generally be applied without concern for these byproducts^13–16^. Ozone has the additional benefit of being more efficient than chlorine in coagulation, a process commonly used for removing organic matter from natural waters during drinking water treatment^11^. High installation and maintenance costs, bulky equipment, and the short half-life of ozone in water have limited the adoption of ozone disinfection. Over the last decade, it has become more popular around the world and deemed one of the best available alternatives to chlorine in water disinfection^17–20^.

Plasma reactors employing corona discharge or dielectric barrier discharge (DBD) are the most common ozone generation devices currently available^21,22^. However, they are limited by high electrical power consumption, low ozone production and expensive-bulky equipment^21,22^. Plasma generation produces ozone by ionizing oxygen in the surrounding gas^22^. In particular, DBD devices allow production of low temperature plasma in air with generation of significant amounts of ozone. DBD plasma is produced when a potential difference is applied across two electrodes on opposite sides of a dielectric (insulator) material. This leads to the formation of filamentary micro discharges and ionization of the surrounding gas^22^. Therefore, ozone generation is an innate characteristic of DBD plasma devices when operated in air. A detailed study relating different parameters such as the applied voltage, frequency and material properties to ozone production using DBD in atmospheric air and its efficacy in killing surface microbes were reported recently by our group^23,24^. In this paper, we use a compact, lightweight DBD plasma generator to test its ability to decontaminate water in an open system. It is run at atmospheric conditions and uses air as a feed gas. This reduces additional costs related to controlled gas conditions like pressure, temperature and oxygen content making it a relatively inexpensive method of ozone generation^25,26^. We used an open system to see if the large closed mixing tank used by many systems could be eliminated to further decrease the cost and size of the ozonizing equipment. Our group has previously used similar plasma generators for killing surface microbes with particular attention to the effect of power and the role of ozone^23,24^.

There are multiple reports devoted to understanding the kinetics of ozone inactivation of microbes and many of them use *Staphlylococcus aureus* and/or *Pseudomonas aeruginosa*^27–32^. In 2009, Zuma *et al*. used corona discharge in an oxygen stream to produce ozone and mixed it in water using an impinger^30^. Inactivation of *P. aeruginosa* in water was also investigated by Lezcano *et al*.^28^ and Pérez *et al*.^29^. However, the details about the ozone generating systems were not given in these papers. Also, sufficient information could not be found regarding minimal ozone concentration requirement for microbial inactivation and related energy consumption for ozone production. These reports and others would be improved by providing more complete experimental details of ozone concentration measured in water and the corresponding exposure time and input energy.

This study establishes the effectiveness of a compact, lightweight DBD plasma reactor in generating ozone and decontaminating water in an open system with air as the oxygen source. We determined the minimal ozone concentration along with exposure time and input energy required for inactivation of *P. aeruginosa* and MRSA. Additionally, this is an attempt to fill the knowledge gap associated with details on ozone generation techniques, ozone mixing processes, ozone concentration in water and power requirements for achieving bacterial inactivation in existing literature on inactivation kinetics of bacteria in water^28–31^.

## Materials and Methods

### Preparation of cultures

Gram-negative (*P. aeruginosa*) and Gram-positive (*S. aureus*) bacteria were selected as test organisms for disinfection experiments. These organisms have been used in previous ozone disinfection studies. They are potential pathogens spread in contaminated water, and are relatively resistant to disinfection. We used recent clinical isolates of *P. aeruginosa* and methicillin-resistant *S. aureus*. Stocks were stored at −80 °C in LB (Luria-Bertani) broth with 30% glycerol. Frozen stocks were grown overnight on LB agar plates at 37 °C. Fresh cultures were diluted in LB broth to give approximately 5 × 10^6^ CFU (colony forming units)/mL and 0.1 ml of the culture was inoculated into 125 mL of distilled deionized water.

### Plasma Generation

**Figure 1:**
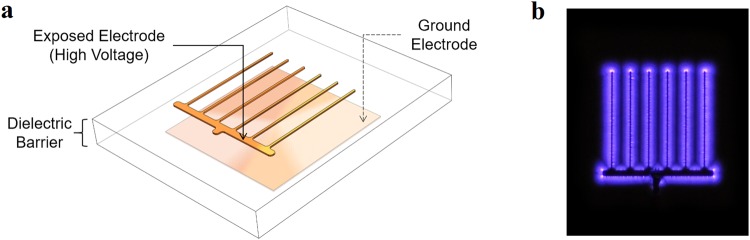
Illustration of the Surface Dielectric Barrier Discharge (DBD) comb-reactor. The image shows (**a**) a diagram of the DBD plasma reactor showing the exposed electrode and the ground electrode separated by a dielectric of 0.76 mm thickness and 3.48 dielectric constant and (**b**) plasma formed around the exposed electrode when the reactor is powered.

Figure 1 illustrates the electrode design used to produce ozone for this study. In this configuration, plasmas are formed over the surface of the dielectric barrier and along the perimeter of the top electrode that is exposed to the surrounding air. Figure 1a shows two copper electrodes placed on the top and bottom surfaces of the dielectric material. The top electrode is a comb-like structure, whereas the bottom electrode has a square shape. The thickness of the copper used in both electrodes is 35 µm. The DBD plasma is generated by applying an alternating potential difference between the two electrodes. Figure 1b shows plasma formation around the top (comb) electrode that is connected to the higher potential and exposed to air. The bottom electrode is grounded and covered by a layer of Kapton tape; therefore, plasma is not formed on the bottom side of the reactor. The dielectric material was a hydrocarbon/ceramic (RO4350B™^33^) composite with a thickness of 0.76 mm and dielectric constant of 3.48. A more detailed description of the comb reactor design can be found in a recent publication by our group^23^.

### DBD power supply circuit

**Figure 2:**
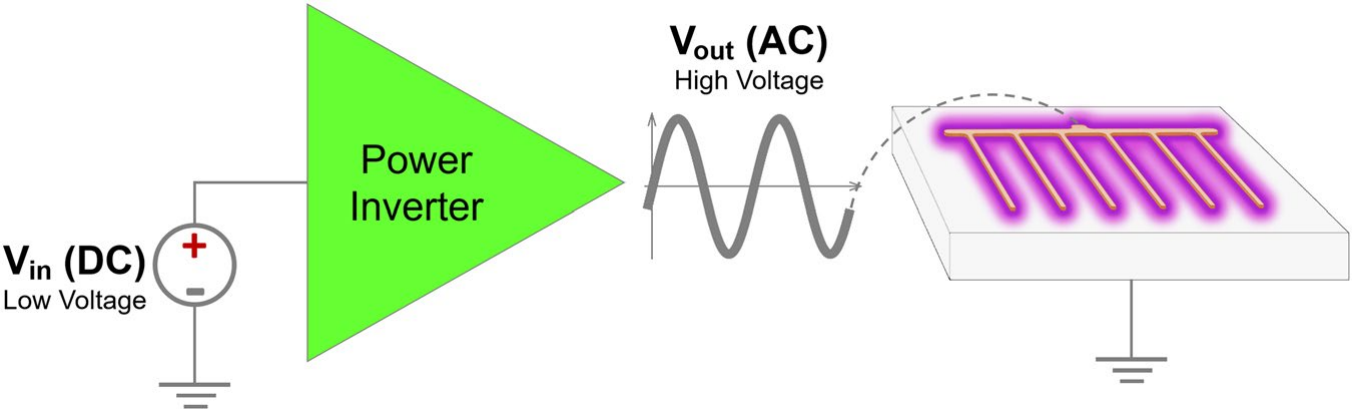
Electrical setup used to generate DBD plasma. A low DC voltage is converted to a high AC voltage using the power inverter. The resulting high AC voltage powers the DBD plasma reactor that produces plasma and generates ozone.

As mentioned previously, SDBD plasma reactors, like the one described in this work, require a high alternating voltage to function. Sources that provide such large voltages are generally heavy (kilograms), bulky and expensive^29,30^, making it impractical to implement them in real-world applications. In contrast, the active plasma module (APM)^34^ used in our experiments is only 48 cubic centimeters and weighs 55 grams. This offers a unique advantage in terms of size, weight, and adaptability to the experimental environment. APM operates as a power inverter that converts a low DC input voltage into a high AC output voltage, which is then used to power the plasma reactor electrodes shown in Fig. 1. The electronic module was configured to work with an input voltage of 25 V (DC) proportioned by adjustable power supply model KORAD KA6005D. These input conditions generate approximately 7 kVpp (peak to peak voltage) at the output. A graphical explanation of this electrical system is presented in Fig. 2.

### Experimental set up

**Figure 3:**
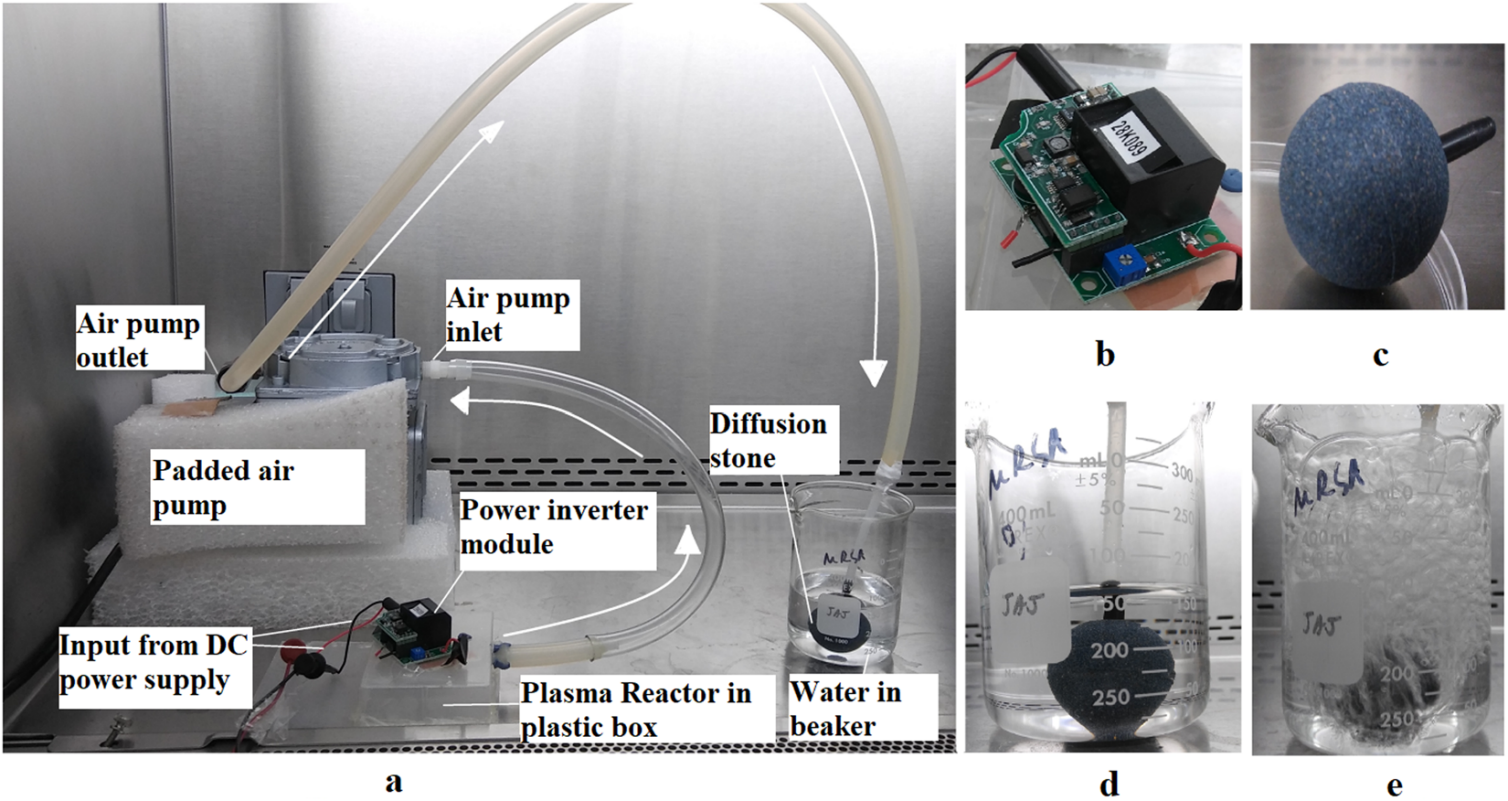
(**a**) Complete experimental set up with arrows showing the ozone flow direction, (**b**) APM power inverter module, (**c**) close up picture of diffusion stone used, (**d**) diffusion stone in contaminated water when pump is OFF, (**e**) diffusion stone in contaminated water when pump is ON.

The experimental set up comprised of a HYVAC PressoVac diaphragm vacuum pump (97000-001 PressoVac 24 Diaphragm Vacuum Pump^35^) with a flow rate of 1 cu ft/min which was used to transfer ozone produced by the plasma reactor to a beaker containing distilled-deionized water, as shown in Fig. 3a–e. Distilled de-ionized (DI) water was used in all experiments to avoid interference of any disinfectant or minerals that might be present in tap water. For decontamination experiments, distilled DI water was contaminated with approximately 5 × 10^5^ CFU/mL of the test organism. The plasma reactor was placed in a small plastic box connected to the vacuum pump to prevent loss of ozone to the atmosphere. The ozone generated was diffused into the water with a 4 cm diameter diffusion stone producing micro bubbles (100 to 500 μm). Experiments were run inside a biosafety cabinet to avoid outside contamination or exposure to bacterial aerosols. All the experimental runs for this study were performed under atmospheric conditions.

### Active ozonation and pre-ozonation of water

The first set of experiments dealt with active ozonation of contaminated water. 0.1 ml of the bacterial culture was mixed with 125 ml of distilled de-ionized water in a sterile beaker to obtain a consistent control concentration of 10^4^–10^5^ CFU/ml for each run. This will be referred to as the control bacterial culture throughout the paper. Ozone was diffused into the beaker and water was sampled at different time points after ozone treatment in sterile tubes. The second set of experiments dealt with pre-ozonated water. Ozone was first diffused into 125 mL of distilled (DI) water for different time periods. This was followed by mixing the ozonated water with 0.1 ml of the bacterial culture. This mixture was left to sit for 30 minutes and sampled. At the end of this period, plate counts were used to determine the effect of pre-ozonated water on bacterial survival. After every experiment, the beaker and the diffusion stone were sterilized with 70% isopropyl alcohol, rinsed with distilled deionized water and dried to prevent erroneous results.

### Determination of disinfection

Concentration of culturable bacteria before and after treatment was determined by the spread plate method. 100 µL of serial dilutions of water were plated on LB agar and incubated at 37 °C for 24 hours before counting^36^. Detection limit of the spread plate method was 10 CFU/ml based on the volume of water tested.

### Ozone measurements in water and air

Hach 2064400 oz-2 Color Disc Test Kit was used to measure dissolved ozone water. The test kit is based on the DPD (N,N-diethyl-p-phenylenediamine) colorimetric method. The reaction of ozone with potassium iodide (KI) results in the formation of iodine which then reacts with DPD forming a pink compound. The intensity of the pink color is an indication of the ozone concentration^37^. The lower and upper limits of sensitivity for the test kit are 0.05 mg/L and 2.2 mg/L, respectively^38^. The DPD colorimetric method has been used successfully to measure ozone in previous research studies^39,40^. The 2B Technologies Model 202 Ozone Monitor™, which works based on UV light absorption at 254 nm, was used for the ozone measurements in air. The accuracy of the monitor was 1.5 ppb or 2% of the reading^41^.

### Power measurements

To estimate the average power consumption of the plasma reactor, an oscilloscope (Tektronix DPO 3014) with recording length set to 1 million points was programmed through a LabVIEW code to collect the voltage and current waveform data every 40 seconds for 4 minutes. The experiment was repeated at least six times. The collected information was processed in MATLAB to obtain the average power and the uncertainty in a 95% confidence interval.

### Control experiments

#### Control experiments to correct bacterial count

At least three repeats of bacterial count measurement in the uncontaminated distilled DI water contained in sterilized beakers without ozonation/pre-ozonation and contamination were carried out initially before starting any experiments to correct for actual inactivation at the end of all data collection. Control experiments were also performed to account for possible growth or inactivation of bacteria in distilled DI water over the length of time of the experiments by comparing the bacterial count of the same contaminated water plated in intervals of 5 and 10 minutes.

#### Control experiments to correct ozone measurements

Ozone concentration was measured in distilled DI water without ozonation/pre-ozonation and any bacterial contamination. Ozone measurements were also taken at different time points during active ozonation of distilled DI water with and without bacterial contamination to correct ozone measurements.

## Results

### Initial Control experiments

#### Correction for bacterial count

Measurements in distilled (DI) water before ozonating/pre-ozonating or mixing bacterial species in it indicated undetectable levels of culturable bacterial concentration (<10 CFU/ml). These measurements were taken prior to starting every experiment. No growth or inactivation of bacteria in distilled DI water over the length of the total time of the experiments (5 and 10 minutes) was observed.

#### Correction for ozone concentration

No ozone concentration (<0.05 mg/L) was detected in distilled DI water before ozonation/pre-ozonation. These measurements were taken prior to every experiment and the results were as expected because of using distilled DI water^42^.

### Active Ozonation

In case of active ozonation, the bacteria were first exposed to ozone for 5, 10 and 15 minutes to observe the killing achieved for each time. Three repeats at each time point (t = 5, 10, 15 min) for initial bacterial concentration of 10^4^–10^5^ CFU/ml were performed and bacterial inactivation to undetectable levels was observed in all the cases. At 5 minutes, 4 to 5 log reduction in the concentration of both bacteria implied that the minimum time of exposure required for deactivation of these bacterial colonies was <5 minutes. Thus, in the following experiments, the contaminated water was exposed to ozone and sampled every minute from t = 1 min to t = 5 min and finally at 10 minutes. Four to five repeats were performed for each time point. Bacterial concentrations calculated for all the repeated experiments can be found in Supplementary Tables 1 and 2. 

**Figure 4:**
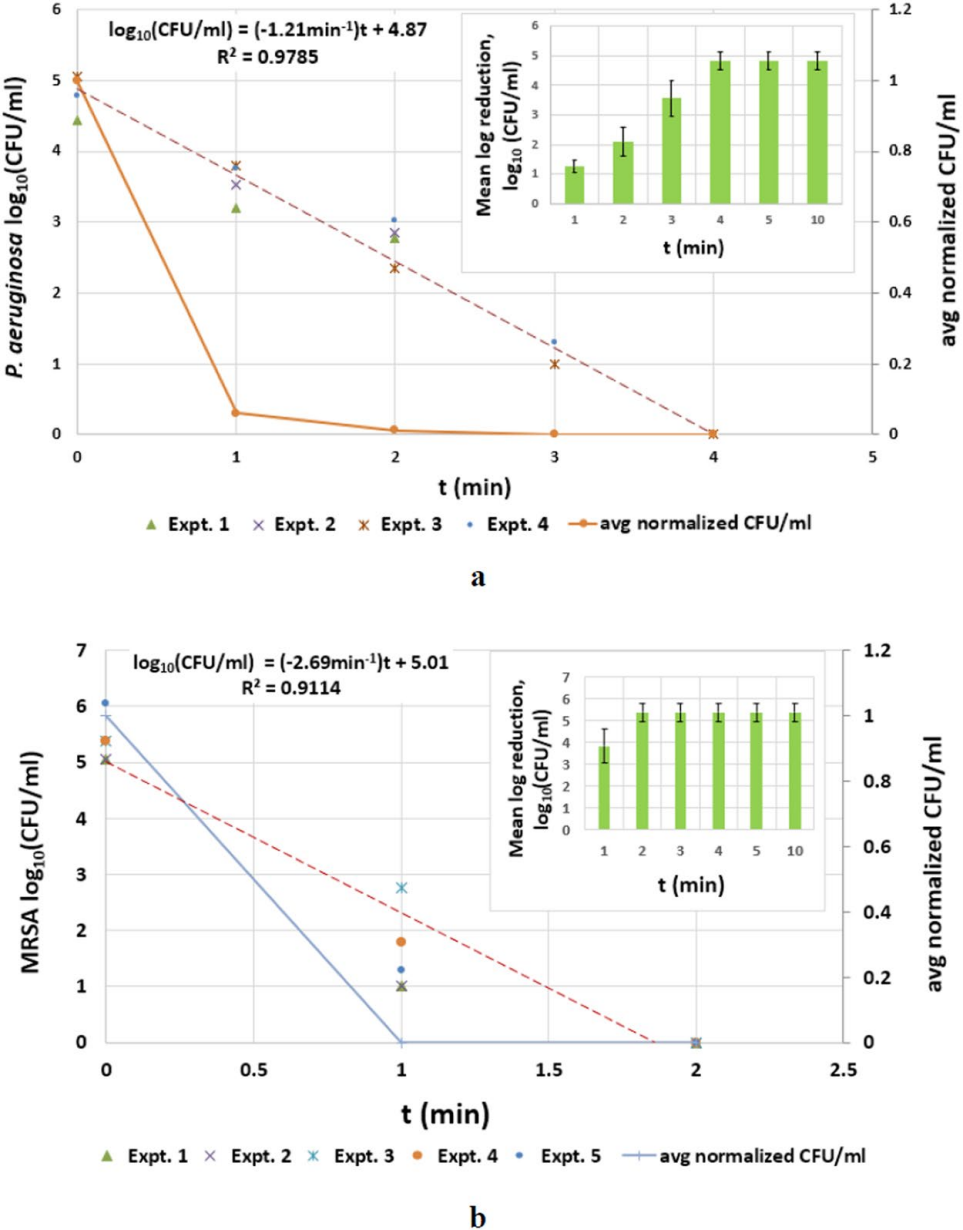
(**a**) Survival curves of *P. aeruginosa* in contaminated water for repeated active ozonation experiments (left axis), average normalized CFU/ml (right axis) and mean log reduction (right top) (**b**) survival curves of MRSA in contaminated water for repeated active ozonation experiments (left axis), average normalized CFU/ml (right axis) and mean log reduction (right top).

The mean logarithmic reduction obtained for each species at each time point was plotted to find the minimum exposure time required to get undetectable levels of bacteria. Error bars for the mean graphs were calculated and plotted based on the standard deviation. From Fig. 4a (right top) it can be observed that an exposure time of 4 minutes is enough to get a 4.8 ± 0.3 log reduction of *P. aeruginosa*. For MRSA, 2 minutes was found to be the minimum exposure time to get a 5.4 ± 0.4 log reduction (Fig. 4b (right top)).

The concentrations of both the bacterial species were followed in actively ozonized water until they were undetectable by plate count (Fig. 4(a,b)). Linear trends were observed in the semi log kill curves for both bacterial species with high R- squared values indicating good fit of the trend lines. This suggests an exponential decay (CFU/ml $\propto$ exp (−t)) of both bacteria with increase in time. Due to the differences in initial bacterial concentration for different repeats of experiments, normalized CFU/ml (concentration at time t/initial concentration) was used to calculate an average value over all the repeats for both bacterial species and is also shown in Fig. 4(a,b) (right axis).

### Pre-ozonation

Pre-ozonated water has been reported to be an effective method for disinfecting water. We diffused ozone from the plasma generator into water using a diffusion stone for different lengths of time: 5, 10, 15 and 20 minutes. The ozonated water was mixed with 0.1 ml of the bacterial culture and held at room temperature for 30 minutes. At the end of this period, plate counts were used to quantify bacterial inactivation. The log reduction observed was negligible irrespective of the time for which the water was ozonated. Even with high exposure times of 10, 15 and 20 minutes; the log reduction obtained was less than 1 log. Factors responsible for this difference between active ozonation and pre-ozonation could be the flow rate at which ozone is diffused into water and the rapid decomposition of ozone in water^30,43^. Moreover, use of an open beaker allowing the escape of ozone being mixed in water is likely to be a factor leading to the differences in active and pre-ozonation disinfection results. This suggests that active ozonation would be better for treatment of open water systems compared to pre-ozonation in the absence of controlled conditions or ozone stabilizing chemical additives. This has been further analyzed in the Discussion section of this paper.

### Ozone measureme**n**ts in air and water

Ozone concentrations in water corresponding to active ozonation experiments were calculated as the difference in measurements taken during active ozonation of (a) 125 ml distilled DI water plus 0.1 ml LB (bacterial culture media) and (b) 125 ml distilled DI water plus 0.1 ml bacterial solution. This takes care of eliminating any ozone demand of distilled DI water and LB. Ozone measurements for all repeated experiments in water with bacteria gave undetectable ozone concentrations (<0.05 mg/L) at all time points till undetectable levels were obtained for both bacterial species indicating complete usage of any dissolved ozone in water by the bacteria.

**Figure 5:**
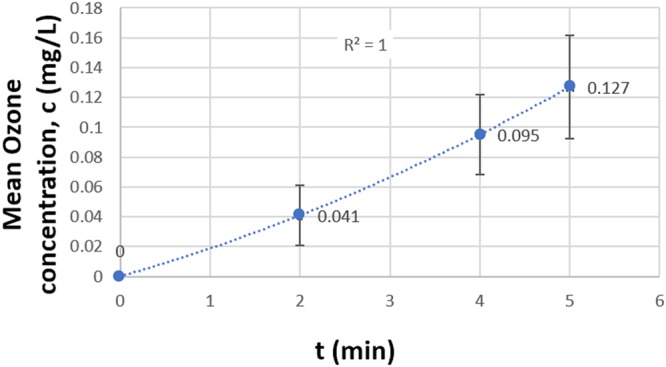
Average ozone measurements in 125 ml distilled DI water plus 0.1 ml LB during active ozonation at time points, t = 2, 4 and 5 minutes calculated from 11 measurements at each time point.

Figure 5 shows the average ozone concentrations in distilled DI water plus LB without bacteria for time points 2, 4 and 5 minutes. 11 measurements were taken at each time point by sampling the water while ozone was being diffused in the water. These measurments can be found in Supplementary Table 3.  It was observed that most of the measurements for 2 minutes gave an ozone concentration of 0.05 mg/L which is the lower limit of detection of the ozone test kit used in this study. A second order polynomial trendline was used to fit the curve. The average dissolved ozone concentration data at 2, 4 and 5 minutes were 0.041 + 0.020, 0.095 + 0.027 and 0.127 + 0.034 mg/L, respectively.

The CT (concentration × time) values were calculated from the ozone concentration data and inactivation time^44^. The CT value for a 4.8 log inactivation of *P. aeruginosa* in water was 0.4 mg-min/L and that for 5.4 log inactivation of MRSA was 0.1 mg-min/L. These values are comparable to the CT values of 0.05 and 0.3 mg-min/L obtained in previous studies performed on 3.5 to 5 log reduction in *E. coli* contaminated water and laboratory waste water using ozone^45–48^. However, the CT values cannot be directly compared to previous studies because of our water system being an open system.

**Figure 6:**
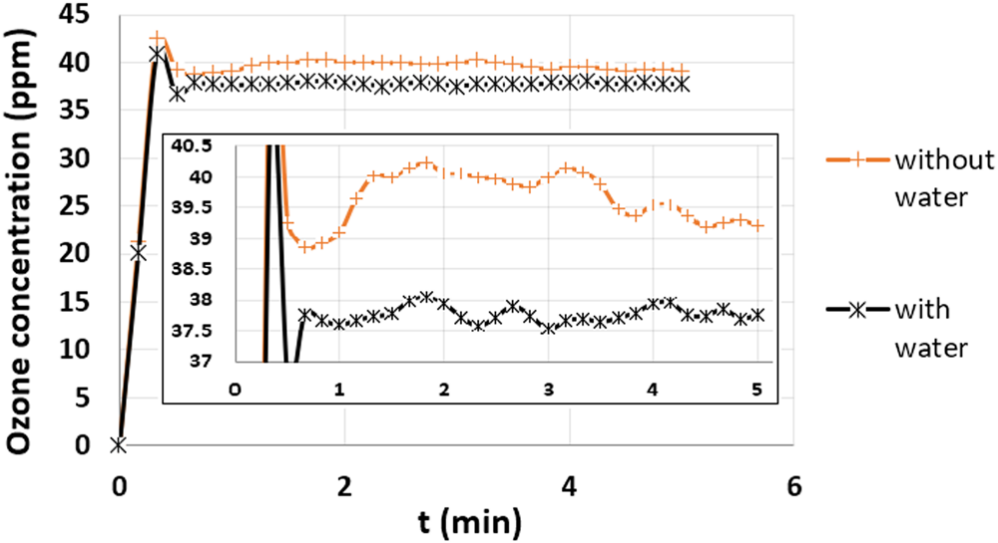
Ozone measurements in the air above the water level with and without water in the beaker. The difference between the two curves is due to the amount of ozone dissolved in the water. The small difference in the two curves is consistent with the low concentration of dissolved ozone in water by direct measurement shown in Fig. 5.

Ozone measurements were also taken in the air right above the water level for 5 minutes in the beaker with and without water in it, while ozone was being diffused through the diffusion stone, as shown in Fig. 6. There are at least four processes taking place when water is present in the beaker that contribute to the final ozone concentration in the air above the water level: (a) ozone dissolving in water, (b) un-dissolved gaseous ozone escaping from water, (c) ozone decomposing in water and (d) ozone decomposing in air. Due to the system being open, ozone concentration in air cannot be directly related to dissolved ozone concentration in water. The volume of air is not fixed in an open system and hence the mass of ozone cannot be directly calculated from the concentrations in air. However, the difference between ozone concentrations in the beaker with and without water can still help explain the small amounts of dissolved ozone measured in water. As shown in Fig. 6, it takes about 0.5 minutes for the ozone concentration in air to come to an equilibrium with respect to all the active processes. When the beaker was empty, the average ozone concentration from 0.5 to 5 minutes was 39.72 ± 1.04 ppm (0.085 ± 0.002 mg/L) compared to 37.87 ± 0.60 ppm (0.081 ± 0.001 mg/L) when there was water in the beaker. The small difference in these two values is consistent with the low concentration of dissolved ozone in water. The following conversion was used to calculate the ozone concentration in air and water, respectively^49^.

$$1\,{\rm{ppm}}\,{\rm{of}}\,{\rm{ozone}}\,{\rm{in}}\,{\rm{air}}=2.14\times {10}^{-3}\,{\rm{mg}}/{\rm{L}};\,1\,{\rm{ppm}}\,{\rm{of}}\,{\rm{ozone}}\,{\rm{in}}\,{\rm{water}}=1\,{\rm{mg}}/{\rm{L}}.$$

For the pre-ozonation experiments, the water was first diffused with ozone for exposure times, t = 5, 10, 15 and 20 minutes^49^. The resulting ozonated water was left to sit for 30 minutes and was sampled at every 5 minutes within this period. For exposure times of 10, 15 and 20 minutes the initial ozone measurements were beyond the scope of the technique used to measure ozone. However, it was observed that no matter what the initial ozone concentration was at the start of the 30 minutes, it dropped to ≤ 0.05 mg/L in 5 minutes and 0 mg/L by 15 minutes.

### Power measurements and ozone yield

**Figure 7:**
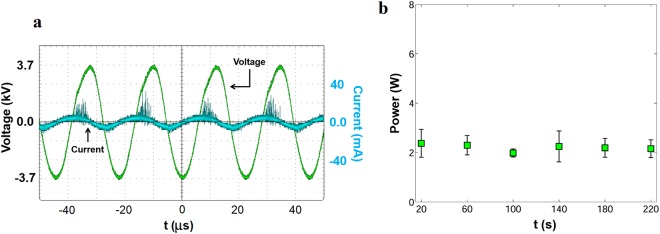
(**a**) Characteristic voltage and current waveforms of the DBD plasma reactor used in this study. (**b**) Average power required to run the DBD plasma reactor is showed for different times. The graph shows a fairly constant value of power around 2.2 ± 0.37 Watts.

Figure 7a shows the voltage and current waveforms detected on the surface DBD reactor during plasma formation. The current waveform is characterized by large current spikes during the positive-going cycle due to the formation of streamers extended through the dielectric. During the negative-going cycle, the plasma morphology corresponds to glow discharges rather than streamers. In this regime, the current spikes are small, but they are more densely populated than the current spikes of the positive-going cycle^50^. Figure 7b shows the average power versus time corresponding to voltage and frequency of 7.4 kV and 45 kHz, which are the values provided by the inverter module. It is observed that the average power remains fairly constant around 2.2 ± 0.37 Watts. Thus, the energy required to run the reactor for 2 and 4 minutes was calculated to be 4.4 ± 0.74 J and 8.8 ± 1.48 J, respectively. An output voltage of 7.4 kV and 45 kHz requires 25 volts (DC) at the input of the power inverter module.

The ozone yield of the SDBD comb reactors using 0.76 mm thick hydrocarbon/ceramic as dielectric material was calculated from the data presented in a recent publication by our group^23^. The maximum ozone yield calculated for the two operating conditions of voltage/frequency of 7 kV/10KHz and 8.5 kV/14KHz was found to be 59.2 and 68.6 g/KWh, respectively.

## Discussion

Ozone produced by the DBD plasma generator resulted in a 5 log drop to undetectable levels of *S. aureus* and *P. aeruginosa* within 4 minutes in distilled-deionized water being actively ozonized. Aqueous ozone concentration of about 0.1 mg/L corresponding to 4 minutes exposure time and 8.8 ± 1.48 J input energy resulted in 4.8 ± 0.3 log reduction of *P. aeruginosa. S. aureus* was slightly more sensitive with no colonies growing after 2 minutes exposure time. 5.4 ± 0.4 log reduction in *S. aureus* was obtained at an ozone concentration and input energy of 0.04 mg/L and 4.4 ± 0.74 J, respectively.

According to a study performed by Zuma *et al*. (2009), 2 minutes exposure time resulted in 1 log reduction in *P. aeruginosa* during active ozonation of contaminated water with an initial bacterial concentration of 10^6^ CFU/ml. The corresponding ozone concentration in water derived from the ozone concentration in the oxygen stream at the given flow rate was found to be 0.9 mg/L^30^. For the same exposure time of 2 minutes our study shows >2 log reduction (Fig. 4) with about 1/10^th^ of the dissolved ozone concentration at 0.1 mg/L (Fig. 5) with similar initial bacterial concentration. In spite of differences in experimental methods used by Zuma *et al*. like using corona discharge for ozone production and an impinger for ozonation of water, our results were fairly similar with respect to exposure time and the resulting log reduction. However, the ozone concentration results varied by a factor of 10. Possible reason for the difference in ozone concentrations may be due to the over-estimation of ozone concentration by calculating it indirectly from the ozone concentration in the oxygen stream at the given flow rate or the difference in ozone consumption by contaminated water in an open and close system. The energy or power required for ozone production was not reported in this study.

Lezcano *et al*. (1999) achieved 4 log reduction of *P. aeruginosa* in 5 minutes with 0.85 mg/L ozone in water for an initial bacterial concentration of 10^4^ CFU/ml. The source of or the energy used for ozone production was not mentioned^28^. With a similar initial bacterial concentration, the corresponding exposure time in our study is 1 minute less and the required dissolved ozone concentration is almost 1/10^th^ of 0.84 mg/L (Figs 4 and 5). These differences may possibly be attributed to overestimation of dissolved ozone by Lezcano *et al*. as the method of ozone measurement in water or ozone demand of the medium was not specified.

Restaino *et al*. (1995) obtained a 4.8 log reduction in 2 minutes for *S. aureus* and a 5.2 log reduction instantaneously for *P. aeruginosa*; both with an initial concentration of 10^6^ CFU/ml^31^. The source of or the energy used for ozone production was not specified. Direct measurement of dissolved ozone in water (i.e. not derived from gaseous ozone concentration) with the indigo colorimetric method was used. The calculated dissolved ozone concentration values at the inlet and outlet of a concurrent system were reported as 0.188 mg/L and 0.064 mg/L, respectively^31^. Our results are similar, confirming a low dissolved ozone concentration is capable of inactivation.

While there are other papers with multiple studies about bacterial inactivation using ozone, we selected the above three papers for comparison since they have the greatest similarity with the methods employed in our study. While our results are broadly similar to previous literature, the prior reports on this subject lack sufficient details with respect to the mixing method, energy consumption and ozone measurement technique that may have resulted in inaccurate ozone concentration estimates. With the highly variable ozone measurements in ozonation systems used previously, it can be asserted that direct measurements of dissolved ozone in water is crucial to finding the minimal ozone concentration for inactivation of bacteria in contaminated water. Moreover, the method used for mixing ozone in water plays an important role in determining the minimum exposure time required. This is because the ozone interaction with bacteria in contaminated water and the reaction time available before the ozone decomposes is greatly dependent on how the mixing occurs.

The results of the pre-ozonation experiments indicated insignificant levels of disinfection and very low ozone concentrations compared to that of active-ozonation for both bacterial species as stated in the Results section. From the ozone measurements in water after the ozone was diffused in water for different exposure times, it was observed that the ozone concentration dropped to 0.05 mg/L within 5 minutes and to 0 mg/L in 15 minutes irrespective of the initial exposure time used to ozonize the water. The ozone concentrations previously reported for 4 to 5 log reduction of *P. aeruginosa* and MRSA in treatment of closed water systems vary from 0.064 to 0.9 mg/L^28,30,31^. Based on these reports, although ozone concentrations of <0.05 mg/L can cause some disinfection, it will not lead to significant reduction in bacterial concentrations. This justifies the less than one log reduction in bacterial species observed in the pre-ozonation experiments conducted in this study. While our DBD generator produced sufficient ozone, the low concentration of dissolved ozone in pre-ozonated water might be explained by the detrimental effects of high flow rate and rapid decomposition of ozone in water^30,43^. A lower flow rate might lead to more mixing and reduce the already low measures of minimum input energy required by the plasma reactor to produce enough ozone for inactivation of bacteria^30,43^. According to Henry’s law, solubility of ozone in water increases with the increase in partial pressure of ozone above it. This suggests that an open system may not be a viable option and more involved ozonizing arrangement with a closed tank and additional power will be required. Alternatively, ozone stabilizers and low temperature could be used to increase the stability of ozone in water^51^. Additional work will be needed to optimize the level of ozone dissolved in water in pre-ozonation applications.

The input energy required to produce ozone for bacterial inactivation to undetectable levels was not specified in most of the research papers on ozone disinfection of contaminated water^27–32^. Perhaps because the primary focus was on understanding the kinetics involved in ozone reactions with bacteria rather than the ozone production. In our paper, we have identified the source of ozone and the energy required to produce adequate ozone as an important factor in water decontamination. The experimental data gathered on the characteristic voltage and current waveforms (Fig. 7a) gives an in depth understanding of the plasma formation leading to ozone production. The plasma morphology in the positive and negative cycle helps to get an insight into how the plasma is formed by the DBD plasma reactor used in this study. The average power data, as shown in Fig. 7b, remains constant at 2.2 ± 0.37 Watts. The product of the power and the time required for inactivation to undetectable levels is the input energy required to produce necessary ozone concentrations in air to be dissolved in contaminated water. In this study, input energy requirements for ozone production using surface DBD plasma reactor required to obtain 4.8 log reduction of *P. aeruginosa* and 5.4 log reduction of MRSA in contaminated water was found to be 8.8 ± 1.48 J and 4.4 ± 0.74 J, respectively while using a diffusion stone for mixing ozone in water.

The maximum ozone yield or ozone production efficiency calculated for the DBD plasma reactor system used in this study was found to be 68.6 g/KWh when the reactor was run at 7 kV and 10 KHz. The ozone yield found is similar to other studies on ozone production by corona and DBD plasma reactors in oxygen or air (10 to 150 g/KWh)^52–56^. Milan *et al*. (2002) reported a maximum ozone yield of 55 g/KWh using corona discharge in synthetic air (20% O_2_ and 80% N_2_)^56^. Koichi Takaki *et al*. (2008) studied different electrode designs for DBD plasma reactors generating ozone in pure oxygen and reported maximum ozone yields of 80 to 120 g/KWh^57^. However, the ozone yield values cannot be directly compared as they depend on various factors like pressure, temperature, the gas used (air, dry air or oxygen), the discharge conditions such as gap-width, applied voltage, its waveforms, reactor size, electrode configuration and dielectric material^57^. Ozone generated using oxygen as feed gas compared to air can double the ozone concentrations produced but increases the cost related to installing and maintaining pure oxygen feeds^58^. Moreover, the chamber size where ozone is produced has been found to affect the ozone measurements which leads to different ozone yield values^59^. Therefore, even though the ozone yield values cannot be compared directly to other studies, the DBD plasma reactor used in our paper can produce adequate ozone concentrations for inactivation with ozone yield similar to other reported studies. Additionally, it can be more economical compared to ozone production systems run in controlled conditions using pure oxygen, dry air or synthetic air as feed gas.

Thus, this paper shows that active ozonation using a small, light weight DBD plasma reactor in atmospheric conditions for ozone production results in 4 to 5 log reduction of *P. aeruginosa* and MRSA in 4 minutes or less with relatively low power use. Using DBD plasma reactors to generate ozone in atmospheric conditions is advantageous because there are no external sources needed to control temperature and pressure conditions. Moreover, they use the oxygen in air to produce ozone eliminating the need to have a supply of pure oxygen tanks. These DBD reactors can also be run with the power supplied by commercial batteries that can provide 25 V DC voltage. These factors make this technology extremely appealing for portable applications involving water decontamination. Further study of inactivation of different bacterial species and the corresponding dissolved ozone and energy requirements for different sized water systems using this technology is required before it can be applied to real world water decontamination processes.

## Conclusions

The effectiveness of an economical and compact atmospheric DBD plasma reactor as an ozone generator for decontamination of water in an open system was investigated. Dissolved ozone concentration in water, exposure time, and input energy required to produce adequate ozone concentrations were identified as significant factors contributing to optimal and effectual inactivation. Therefore, these factors will play a crucial role in improving water purification by ozone. In addition, direct measurement of dissolved ozone in water was found to be critical for understanding the ozonation experiments and determining the minimal ozone concentration and input energy required for bacterial inactivation.

The ozone concentration required for 4 log reduction of *Pseudomonas aeruginosa* and 5 log reduction of methicillin-resistant *Staphylococcus aureus* in water was 0.1 mg/L (exposure time = 4 minutes) and 0.04 mg/L (exposure time = 2 minutes), respectively. These results together indicate possible inactivation of other vegetative cells with ozone concentration in the range of 0.04 to 0.1 mg/L. Input energy levels of 8.8 J and 4.4 J to run the plasma reactor was found to be sufficient to produce the required ozone to be mixed in water by a diffusion stone for achieving bacterial inactivation (<10 CFU/ml). A maximum ozone yield of 68.6 g/KWh for the compact and lightweight DBD plasma reactor system was found to be similar with reported ozone yields in previous studies signifying the applicability of the DBD plasma reactor system as an ozone generator in water decontamination processes. However, the pre-ozonation results indicate that it is not feasible to disinfect open water systems with pre-ozonation unless the decomposition of ozone in water is inhibited.

Thus, the ability of the DBD plasma reactor system to produce ozone for water decontamination was examined and effective bacterial inactivation was observed. This paves the way for future applications of this ozone generator as it is a compact system and does not incur expenses related to controlled pressure and temperature conditions or feed gas composition. The kinetics of bacterial inactivation with ozonation using this technology requires further study in terms of obtaining more data points in the early part of the survival curves when there are reduced numbers of culturable bacteria to calculate rates of bacterial inactivation. This should be done in the near future to determine an accurate model for kinetics of inactivation for various bacterial species.

## Electronic supplementary material

Supplementary Dataset 1
