# Supplementary material for: Inactivation of Pseudomonas aeruginosa and Methicillin-resistant Staphylococcus aureus in an open water system with ozone generated by a compact, atmospheric DBD plasma reactor
Source: Sci Rep. 2018 Dec 4;8:17573. doi: 10.1038/s41598-018-36003-0 (PMC6279761; doi:10.1038/s41598-018-36003-0)
Supplement: Supplementary file 1 — Supplementary Dataset 1 [file 41598_2018_36003_MOESM1_ESM.pdf]

Inactivation of *Pseudomonas aeruginosa* and Methicillin-resistant *Staphylococcus aureus* in an open water system with ozone generated by a compact, atmospheric DBD plasma reactor

Bhaswati Choudhury[1,2], Sherlie Portugal[1,3,4], Navya Mastanaiah[1,2], Judith A. Johnson[1,5,6], Subrata Roy[1,2,7,*]

[1] Applied Physics Research Group, University of Florida, 32611, Gainesville, USA
[2] Department of Mechanical and Aerospace Engineering, University of Florida, 32611, Gainesville, USA
[3] Department of Electrical and Computer Engineering, University of Florida, Gainesville, 32611, USA
[4] School of Electrical Engineering, Technological University of Panama, Panama City, Panama
[5] Department of Pathology, Immunology and Laboratory Medicine, University of Florida, Gainesville, 32611, USA
[6] Emerging Pathogens Institute, University of Florida, Gainesville, 32611, USA
[7] SurfPlasma, Inc., Gainesville 32601, USA

*roy@ufl.edu

Table 1: Results obtained from repeated active ozonation experiments on water contaminated with *P. aeruginosa*. Columns two through eight shows the bacterial counts in Colony Forming Units per milliliter (CFU/ml) for active ozonation experiments at time points, t = 0, 1, 2, 3, 4, 5 and 10 minutes. The Control count is defined as the bacterial count at the start of the experiment (t=0 minutes).

**Expt. No.**	**Control count at t=0 min (CFU/ml)**	**Count at t=1 min (CFU/ml)**	**Count at t=2 minutes (CFU/ml)**	**Count at t=3 minutes (CFU/ml)**	**Count at t=4 minutes (CFU/ml)**	**Count at t=5 minutes (CFU/ml)**	**Count at t=10 minutes (CFU/ml)**
1	$2.7x10^4$	$1.6x10^3$	$5.8x10^2$	10	0	0	0
2	$1.1x10^5$	$3.3x10^3$	$7.1x10^2$	10	0	0	0
3	$1.1x10^5$	$6.3x10^3$	$2x10^2$	10	0	0	0
4	$6x10^4$	$5.8x10^3$	$1.1x10^3$	20	0	0	0

Table 2: Results obtained from repeated active ozonation experiments on water contaminated with Methicillin-resistant *Staphylococcus aureus* (MRSA). Columns two through eight shows the bacterial counts in Colony Forming Units per milliliter (CFU/ml) for active ozonation experiments at time points, t = 0, 1, 2, 3, 4, 5 and 10 minutes. The Control count is defined as the bacterial count at the start of the experiment (t=0 minutes).

**Expt. No.**	**Control count, at t=0 min (CFU/ml)**	**Count at t=1 min (CFU/ml)**	**Count at t=2 minutes (CFU/ml)**	**Count at t=3 minutes (CFU/ml)**	**Count at t=4 minutes (CFU/ml)**	**Count at t=5 minutes (CFU/ml)**	**Count at t=10 minutes (CFU/ml)**
1	$1.2x10^5$	10	0	0	0	0	0
2	$1.1x10^5$	10	0	0	0	0	0
3	$2.4x10^5$	$5.7x10^2$	0	0	0	0	0
4	$2.4 x10^5$	60	0	0	0	0	0
5	$1.1 x10^6$	20	0	0	0	0	0

Table 3. Ozone measurements were taken in water during active ozonation at time points, t= 3, 4, 5 and 10 minutes. The Hach 2064400 oz-2 Color Disc Test Kit was used to take the measurements. The upper and lower limits of detection of the Color Disc were 0.05 mg/L and 2.2 mg/L respectively, with each step on the disc representing an increment of 0.05 mg/L. The average of 11 measurements at each time point was taken to calculate the ozone concentration at a time point.

**Ozone concentration (mg/L)**			
**Trials/ time**	**2 min**	**4 min**	**5 min**
**1**	0.05	0.1	0.1
**2**	0.05	0.1	0.15
**3**	0.05	0.05	0.1
**4**	0.05	0.1	0.1
**5**	0	0.1	0.15
**6**	0.05	0.1	0.1
**7**	0.05	0.15	0.2
**8**	0	0.1	0.1
**9**	0.05	0.05	0.1
**10**	0.05	0.1	0.15
**11**	0.05	0.1	0.15
**Average**	0.041	0.095	0.127
